# Supplementary material for: Amlexanox: Readthrough Induction and Nonsense-Mediated mRNA Decay Inhibition in a Charcot–Marie–Tooth Model of hiPSCs-Derived Neuronal Cells Harboring a Nonsense Mutation in GDAP1 Gene
Source: Pharmaceuticals (Basel). 2023 Jul 21;16(7):1034. doi: 10.3390/ph16071034 (PMC10385573; doi:10.3390/ph16071034)
Supplement: Supplementary file 1 [file pharmaceuticals-16-01034-s001.zip › pharmaceuticals-2445955-supplementary.pdf]

Supplementary Table 1. Antibodies dilutions and references.

Antibody	Dilution	Reference
GDAP1	1:25	Proteintech
Tuj1	1:1000	R&D
Nestin	1:400	Merck
Olig2	1:200	Abcam
